# Complementary Feeding Practices among Mothers in Selected Slums of Dhaka City: A Descriptive Study

**Published:** 2014-03

**Authors:** Farzana Saleh, Ferdous Ara, Md. Asirul Hoque, Md. Safiul Alam

**Affiliations:** ^1^Department of Community Nutrition, Bangladesh University of Health Sciences, Dhaka 1216, Bangladesh; ^2^Plan International Bangladesh, Bangladesh University of Health Sciences, Dhaka 1216, Bangladesh

**Keywords:** Children, Complementary feeding practices, Complementary food, Energy intake, Exclusive breastfeeding, Infant-feeding practices, Infants, Slum, Bangladesh

## Abstract

Improper complementary feeding (CF) practice is one of the main reasons for malnutrition among Bangladeshi children aged less than two years. In this context, using the guidelines of the World Health Organization (WHO), this study assessed the CF practices among mothers in four selected slums (Tejgoan, Rayerbazar, Beribadh, and Jafrabad) of Dhaka city. This descriptive study, conducted during January-June 2010, included 120 mother-child pairs from the selected slums. Samples were selected conveniently, and the sociodemographic profiles of mothers in the four slums were similar. The mean (standard deviation) age of the children was 14.68±5.55 months. A questionnaire, developed following the guidelines of WHO for CF practices, was used for collecting data. Twenty-seven (23%) mothers were exclusively breastfeeding (EBF) their children. Among non-EBF mothers, 15 (16%) started CF after the recommended time. At 6-8 months of age, 2 (40%) of the EBF and 12 (67%) of the non-EBF mothers gave complementary foods twice a day to their children. In both the groups—9-11 months of age—about 70% mothers gave complementary foods twice a day to their children. The frequency of CF was acceptable (3 times a day) in 13 (81%) of the EBF and 32 (56%) of the non-EBF children at 12-23 months of age. Complementary foods given by 24 (89%) of the EBF and 86 (93%) of the non-EBF mothers to their children were not adequate in energy contents. Two (7%) EBF and 16 (17%) non-EBF mothers did not wash their hands after defaecation. Three (11%) EBF and 24 (26%) non-EBF mothers did not properly clean their hands and utensils before feeding. Nine (33%) EBF mothers did not wash their children's hands. Fifty (54%) non-EBF mothers also did not do this. Feeding with psychosocial care practices was not perfect in either of the groups. The findings showed that, according to the WHO guidelines, the CF practices among mothers of children aged less than two years were very poor in the selected slums of Dhaka city. These findings indicate that there is a considerable gap between the recommendations of WHO and the energy intake among this group of children.

## INTRODUCTION

Adequate nutrition during infancy and early childhood is essential to ensure the growth, health, and development of children to their full potential. The World Health Organization (WHO) and United Nations Children's Fund (UNICEF) recommend exclusive breastfeeding (EBF) for six months, i.e. 180 days (1) and addition of complementary foods at six months of age with continued breastfeeding till at least two years (2). Globally, optimal breastfeeding could prevent 13% of deaths of children aged less than five years while appropriate complementary feeding (CF) practices might result in an additional 6% reduction in under-five mortality (3), especially in developing countries as ours. Poor feeding practices, coupled with high rates of infectious diseases, are the proximate causes of malnutrition during the first two years of life. The second half of an infant's first year is especially a vulnerable time when breastmilk alone is no longer sufficient to meet his/her nutritional requirements, and CF should be started (4).

During the period of CF, the young child gradually becomes habituated to eating family foods. Complementary foods bridge the gap in energy, vitamin A, and iron intake, which occurs in breastfed infants at six months of age (2). Too early or too late introduction of CF may lead to nutritional deficiencies of iron, zinc, calcium, and vitamins (2). Therefore, CF needs to be nutritionally adequate and safe and appropriately fed to meet the energy and nutrient needs of the young child.

CF is also influenced by cultural factors, beliefs, and knowledge of parents on appropriate practices (5). Similarly, psychosocial care, safe preparation and proper storage of complementary foods, and hygiene practice are also the important determinants of proper CF practices.

Urbanization is now occurring at a rapid pace, which has significant implications for child survival. Slums in Dhaka are characterized by poor environmental sanitation and livelihood conditions. Generally, the recommended feeding practices are not followed in the urban or rural communities in Bangladesh (6). Moreover, due to limited health services, the recommendations are also not followed in urban slums.

To the best of our knowledge, no studies have been undertaken to gather information on CF practices among slum mothers in Dhaka. In this study, we assessed the CF practices, with reference to the recommendations of WHO, in selected slums of Dhaka city.

## MATERIALS AND METHODS

### Study site and subjects

This descriptive study was carried out during January-June 2010 in four urban slums (Tejgoan, Rayerbazar, Beribadh, and Jafrabad) in Dhaka city. In total, 120 mother-child pairs were selected conveniently. The mean (standard deviation) age of the children was 14.68±5.55 months. The minimum required sample-size was calculated using the formula: n=Z^2^pq/d^2^ where n=required sample-size, p=expected proportion, Z=95% confidence interval, and d=5% error. In Bangladesh, the proportion of CF practices among the mothers of 6-9 months old children is 74% (7). Therefore, p=74% was taken as the expected proportion, i.e. 0.74, and q is (1-p)=0.26. The study included children who were given breastmilk and taking any complementary foods.

### Collection of data

Data were collected through face-to-face interviews, using a questionnaire developed following the guidelines of WHO (2) for CF practices. The sociodemographic profiles of mothers of the four slums were similar.

The dietary intake of the children was calculated using 24-hour recall (8). Adequacy of CF practices was assessed according to the recommendation of WHO (4): 6-8 months: 2-3 meals + 1-2 snack(s) (200 kcal) per day; 9-11 months: 3-4 meals + 1-2 snack(s) (300 kcal) per day; and 12-23 months: 3-4 meals + 1-2 snack(s) (550 kcal) per day.

### Analysis of data

Data were analyzed using the SPSS software (version 11.5). Frequencies were calculated for descriptive analysis.

### Ethical approval

Ethical approval was obtained from the Ethical and Research Review Committee of the Diabetic Association of Bangladesh. Informed written consent was taken from all the mothers after full explanation of the nature, purpose, and all procedures used for the study. Confidentiality was maintained throughout the study period.

## RESULTS

**Figure 1.**
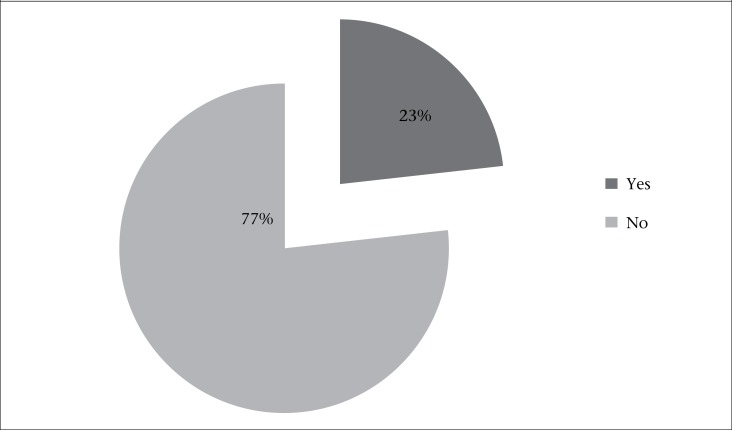
Distribution of mothers according to EBF practices (N=120)

At the time of interview, only 27 (23%) mothers were exclusively breastfeeding their children (Figure 1). The reasons for not exclusively breastfeeding the children were lack of knowledge in 24 (26%) of the cases; about 16 (17%) had no knowledge about the starting age of children for CF; 20 (21%) failed to produce enough breastmilk; and 26 (28%) were sick during the study period (Table 1).

Children of both EBF and non-EBF mothers were divided into three age-groups, viz. 6-8; 9-11, and 12-23 completed months. The distribution of the children among these three age-groups was the same as in EBF mothers: 5 (19%) at 6-8 months; 6 (22%) at 9-11 months, and 16 (59%) at 12-23 months. Similarly, among non-EBF mothers, the distribution of the children was as follows: 18 (19%) at 6-8 months; 18 (20%) at 9-11 months; and 57 (61%) at 12-23 months. Of the non-EBF mothers, 15 (16%) started CF after the recommended time, viz. late initiation of CF; and 78 (84%) started CF before six months of age of the child. About 52% EBF mothers did not give home-made foods whereas 42 (45%) non-EBF mothers had the same practice. At 6-8 months of age, 3 (60%) EBF mothers gave the main meal to their children thrice a day, and 2 (40%) gave twice a day. Four (80%) EBF mothers never gave snacks to their children of this age. Only six (33%) non-EBF mothers gave the main meal thrice a day at 6-8 months of age, 12 (67%) gave it twice a day, and 13 (72%) did not give any refreshment or snacks. Only 2 (33%) EBF mothers gave the main meal to their children thrice a day at 9-11 months of age, and 4 (67%) gave it twice a day, and a similar percentage (67%) never gave snacks to their children. Five (28%) non-EBF mothers gave the main meal thrice a day to their children of the same age, and 13 (72%) gave twice a day. Twelve (67%) mothers did not give any snacks to their children of this age-group. Thirteen (81%) EBF and 32 (56%) non-EBF mothers of 12-23 months old children followed the WHO-recommended meal frequency, i.e. three times daily whereas 9 (56%) and 26 (46%) EBF and non-EBF mothers respectively did not give snacks to their children of this age-group. Complementary foods given by 24 (89%) EBF and 86 (93%) non-EBF mothers to their children were not adequate in energy contents. Only 1 (4%) and 2 (7%) EBF mothers gave complementary foods to their children respectively during illness and after recovery. Similarly, only three (3%) non-EBF mothers gave complementary foods to their children during illness. After recovery, none of the non-EBF mothers gave complementary foods to their children (Table 2).

**Table 1.** Reasons for not giving exclusive breastfeeding (n=93)

Reason	Frequency (%)
Lack of knowledge on exclusive breastfeeding	24 (26)
Lack of knowledge about the starting age of complementary feeding	16 (17)
Do not produce enough breastmilk	20 (21)
Sickness of mother	26 (28)
Others	7 (8)

Table 3 shows the hygiene practices of the mothers in food preparation and during feeding. Two (7%) EBF and 16 (17%) non-EBF mothers did not wash their hands after defaecation. Three (11%) EBF and 24 (26%) non-EBF mothers did not properly clean their hands and utensils before feeding. Washing of the children's hands before feeding was not a popular practice among the slum mothers, and 9 (33%) EBF mothers did not practise it. Fifty (54%) non-EBF mothers also did not wash their children's hands before feeding. Ten (37%) and 49 (53%) non-EBF mothers did not cover foods after cooking. Besides, 10 (37%) EBF and 28 (30%) non-EBF mothers did not reheat the leftover foods before serving. Most (n=117, 98%) mothers followed the proper duration of food preservation.

**Table 2.** Complementary feeding practices

Variable	Frequency (%)
Time of starting complementary feeding (n=93)	
Before 6 months (Early initiation)	78 (84)
At 6 months (Late initiation)	15 (16)
Using family food as complementary feeds (n=120)	

	EBF children (n=27)	Non-EBF children (n=93)
Yes	13 (48)	51 (55)
No	14 (52)	42 (45)
Number of complementary feeds/day (n=120)		

	Frequency of meal pattern			
Age (completed months)	EBF children (n=27)		Non-EBF children (n=93)	
	2 times/day	3 times/day	2 times/day	3 times/day
6-8	2 (40)	3 (60)	12 (67)	6 (33)
9-11	4 (67)	2 (33)	13 (72)	5 (28)
12-23	3 (19)	13 (81)	25 (44)	32 (56)

	Frequency of snacks			
	EBF children (n=27)		Non-EBF children (n=93)	
	1 time/day	Never	1 time/day	Never
6-8	1 (20)	4 (80)	5 (28)	13 (72)
9-11	2 (33)	4 (67)	6 (33)	12 (67)
12-23	7 (44)	9 (56)	31 (54)	26 (46)
Adequacy of energy in complementary feeds (n=120)				

	EBF children (n=27)	Non-EBF children (n=93)
Adequate	3 (11)	7 (7%)
Not adequate	24 (89)	86 (93)
Giving complementary feeds during and after recovery of illness (n=120)		

	EBF children (n=27)		Non-EBF children (n=93)	
	During illness	After recovery	During illness	After recovery
Yes	1 (4)	2 (7)	3 (3)	0 (0)
No	26 (96)	25 (93)	90 (97)	93 (100)

**Figure 2.**
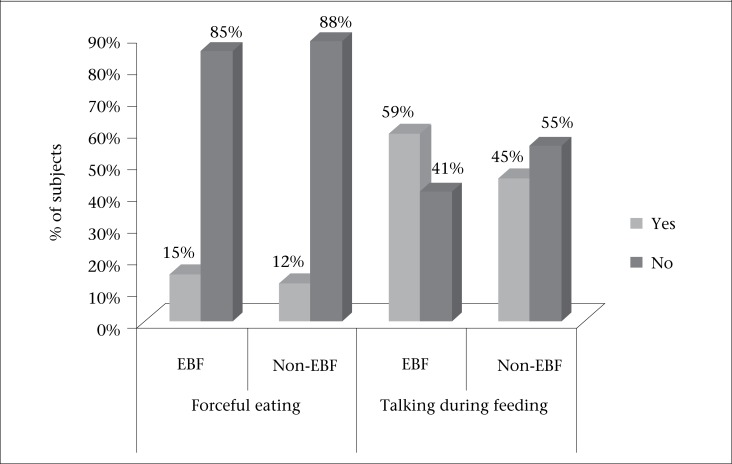
Distribution of mothers according to their psychosocial care during CF practice (n=120)

Four (15%) EBF and 11 (12%) non-EBF mothers forced their children to eat. About 41% EBF and more than half of (55%) the non-EBF mothers did not talk to their children during the feeding time (Figure 2).

## DISCUSSION

The findings of the study revealed that, in Dhaka city, the CF practices among slum mothers of children aged 6-23 months were inappropriate as per the WHO guidelines. According to WHO, exclusively-breastfed children have more rapid growth in the first six months of life than other infants (1). Only 23% of the study mothers practised EBF. Similar results were found in an urban slum of Kolkata (9). In a Delhi urban slum, 35% of mothers practised EBF (10). In a tertiary hospital in India where the rules and care of the newborn baby and mother were followed strictly, 63.5% of mothers exclusively breastfed their infants till six months of age (11).

Two of the 10 study mothers frequently reported that they did not exclusively breastfeed their children because they were unaware of its benefit and had insufficient breastmilk and also because of sickness of some mothers during the study period. Similar reasons about failure to provide EBF were reported from a tertiary hospital in Pakistan (12). It is important for parents to know the short-term and long-term benefits of EBF to both child and mother, including protection of children against various acute and chronic disorders.

**Table 3.** Hygiene practices during food preparation and feeding by mothers

Practice	Frequency (%)	
	EBF children (n=27) No. (%)	Non-EBF children (n=93) No. (%)
Wash hands after defaecation		
Yes	25 (93)	77 (83)
No	2 (7)	16 (17)
Clean hands and utensils before feeding		
Yes	24 (89)	69 (74)
No	3 (11)	24 (26)
Wash hands of children before feeding		
Yes	18 (67)	43 (46)
No	9 (33)	50 (54)
Cover foods after cooking		
Yes	17 (63)	44 (47)
No	10 (37)	49 (53)
Reheat leftover foods before serving		
Yes	17 (63)	65 (70)
No	10 (37)	28 (30)
Duration of preservation of complementary feeds (n=120)		
Proper duration (up to 2 hours)	117 (98)	
Not proper duration	3 (2)	

Results of the review of studies in developing countries showed that infants who are not breastfed 6-10 times daily (13) are more likely to die in the first months of life than infants who are breastfed (14). Children who were breastfed had higher cognitive function compared to those who were formula-fed (15). A study in a Dhaka slum found that deaths from diarrhoea and pneumonia could be reduced by one-third if infants are exclusively, instead of partially, breastfed for the first four months of life (16).

In the present study, only 23% of the mothers started CF at six months of age of their children. A study in the tertiary hospital in India has shown that 17.5% of mothers started CF at the recommended time (11). A study in a tertiary hospital in Pakistan reported that an undesirable early and late introduction of CF was also practised (12). In another study in Delhi slums, 16.6% of mothers started CF at the right time (17), though in our study among non-EBF mothers, similar percentage was found in late introduction of CF. At the same time, 84% of the children were started feeding early. Both these practices are unwanted. Results of a study in Malawi revealed that the early introduction of complementary foods led to problems of infections and malnutrition (18).

In the present study, 48% of the EBF and 55% of the non-EBF mothers started CF with home-made foods. Results of a study in a tertiary hospital in India showed that about 81% of mothers gave home-made foods as complementary feeds to their children (11).

As a child gets older and needs a larger total quantity of food each day, the food needs to be divided into larger numbers of meals. According to the WHO guidelines, EBF children should be given CF 2-3 times a day at 6-8 months and 3-4 times at 9-11 months and also at 12-23 months of age (4). In the present study, the EBF children of each age-group did not receive CF at the recommended frequency, except the children of 12-23 months age-group. In our study, about 40% of the 6-8 months old children received CF twice a day, and 60% of children in the same age-group received CF three times a day. Only 33% of the 9-11 months old children received CF at the recommended frequency of three times a day, although 81% of the 12-23 months old children received CF at the recommended frequency, i.e. three times a day.

In India, 39.3% of mothers gave three or more feeds per day to their children (11). In Pakistan, 50% of 12-23 months old children received CF at the recommended frequency of three or four times a day compared to 21% of 2-3 months old babies who received CF instead of breastmilk, and 19% of 6-8 months old children were only breastfed instead of receiving CF (12).

Most (89%) EBF children in our study did not get adequate amount of energy from CF. In India, only 3.5% of mothers started CF in adequate quantity and with proper consistency (11). The proportion of the non-EBF children was also high (93%) in our study. In the present study, practice relating to the quantity and frequency of CF was poor among most mothers. Incorrect CF practices affect childhood nutrition in developing countries, like Bangladesh. Hence, mothers must have more knowledge about appropriate practices relating to CF for improving the nutritional status of infants, thereby reducing their morbidity and mortality rates.

During illness, the need for fluid often increases; so, a child should be offered and encouraged to take more fluid, and breastfeeding on demand should continue (4). A child should also be encouraged to eat some complementary foods in the illness period to maintain nutrient intake and enhance recovery (19). Intake is usually better if the child is offered his/her favourite foods, and if the foods are soft and appetizing. The amount eaten at any one time is likely to be less than usual; so, the mother may need to give smaller meals more frequently. When the infant or young child is recovering, and his/her appetite improves, the mother should offer an extra portion at each meal or add an extra meal or snack each day. However, in the present study, only 4% and 7% of the EBF mothers gave complementary foods during illness and after recovery respectively.

Meagre data on hygiene practices during food preparation and feeding are available. Results of a study in northwestern Nigeria showed that only 28% of mothers washed their hands before preparing foods, and 19% always sterilized feeding bottles (20). Microbial contamination of CF is a major cause of diarrhoea, which is particularly common among slum children in Dhaka. In the present study, about 7% of the EBF and 17% of the non-EBF mothers did not wash their hands after defaecation. About 11% of the EBF and 26% of the non-EBF mothers did not clean their hands and utensils before feeding. One-third (33%) of the EBF and 54% of the non-EBF mothers did not teach their children how to wash hands before feeding. About 37% and 53% of the EBF and non-EBF mothers respectively never covered foods after cooking. Before serving, 37% of the EBF and 30% of the non-EBF mothers did not reheat the leftover foods. Most (98%) mothers had knowledge about the duration of preservation of complementary foods.

Behavioural studies revealed that responsive feeding or feeding with psychosocial care practices has a positive effect on child's growth and his/her psychological development (21,22). In our study, about 15% of the EBF and non-EBF mothers forced their children to eat. However, more than half (55%) of the EBF mothers talked to their children during feeding, although the proportion was nearly less than half (45%) in the non-EBF children, which was not acceptable.

In the present study, a less proportion of EBF practice, delay in starting CF, improper frequency, and inadequate energy consumption were observed among the mothers. Responsive feeding practice was also not good enough. Based on the findings of the study, we suggest that the health planners and providers extend assistance and essential information to mothers about feeding practices. Besides, research activities in all aspects of CF practices should be emphasized and encouraged.

Due to limited time and resources, we could not organize a larger sample-size to generalize the results of our study for the whole population of Bangladesh. Nevertheless, the findings of the study provide valuable information on the current status of CF practices in the selected slums of Dhaka city.

## ACKNOWLEDGEMENTS

The study was supported by the Bangladesh University of Health Sciences (BUHS) and the Diabetic Association of Bangladesh. We acknowledge our students in the MPH course under the Department of Community Nutrition for their sincere help during data collection.
